# Clinical Case

**Published:** 1895-10

**Authors:** F. G. Davis

**Affiliations:** Quincy, Ill.


### CLINICAL CASE.

F. G. Davis, M. D., Quincy, Ill.

Mrs. R. L., æt. sixty. Thin, tall blonde.

May 4th, 1894.—Appeared for the removal of a wart on face one inch below the right eye. Wart was horny and hard, except the base, which was soft and slightly red.

There was some deafness, with neuralgia in head about temples and sides of face. Unpleasant buzzing in ears. ℞ Caust.$^{1m}$ (F.) dry.

May 11th.—Wart looks the same, but since taking the remedy has suffered very much with rawness between the toes, smarting and itching; bowels became loose. This foot trouble was an old symptom she had got some better of, but it had begun to come on since the warm weather. ℞ Sac-lac.

May 21th.—Reports no better; feet sore; profuse perspiration of feet; used to suffer with herpes on legs. ℞ Kali$^{cm}$ (F.).

June 12th.—Reported wart dropped off several days ago, leaving no mark; feet are better. ℞ Sac-lac.

